# Neonatal Sepsis Caused by Human Parechovirus Type A3 With Marked Hyperferritinemia: A Case Report

**DOI:** 10.1155/crpe/8815738

**Published:** 2025-09-18

**Authors:** Fumihiro Ochi, Mao Niida, Ayumi Sawada, Kozo Nagai, Hitomi Hino, Koji Takemoto, Hisamichi Tauchi

**Affiliations:** ^1^Department of Pediatrics, Ehime Prefectural Central Hospital, Matsuyama, Japan; ^2^Department of Pediatrics, Ehime Prefectural Niihama Hospital, Niihama, Japan; ^3^Department of Infection Control and Prevention, Ehime University Graduate School of Medicine, Toon, Japan

## Abstract

**Background:** In neonates and young infants, human parechovirus A3 (PeV-A3) is associated with severe infections, such as sepsis and encephalomyelitis. However, the mechanisms behind severe illness and the proper indications and methods for treatment remain ambiguous.

**Case Report:** A previously healthy 25-day-old female was admitted to our hospital with a history of high-grade fever, a growling voice, and poor feeding. Upon examination, she appeared lethargic and somnolent, exhibiting symptoms of tachycardia, tachypnea, and peripheral coolness. Sepsis evaluations, including the FilmArray Meningitis/Encephalitis panel, confirmed the presence of PeV-A3 infection. Empirical antibiotic therapy with ampicillin and cefotaxime was started. The fever subsided by Day 4, and a negative bacterial culture indicated that antibiotics were no longer necessary. However, on Day 5, the patient experienced a drop in platelet count, elevated liver enzymes, and hyperferritinemia (ferritin level of 37,223 ng/mL). Despite the high ferritin levels, hemophagocytic lymphohistiocytosis (HLH) was not observed, and the patient was treated without immunosuppressive therapy. Her condition improved, and she was discharged on Day 14. The isolated PeV was genotyped as PeV-A3.

**Conclusions:** PeV-A3 infections often link to hyperferritinemia. Although some studies indicate that steroids and immunosuppressants might be beneficial, this case shows that diligent observation could be adequate, even with high ferritin levels. Monitoring clinical status and lab results to assess whether treatment is necessary is crucial.

## 1. Introduction

Human parechoviruses (PeVs) are small, nonenveloped RNA viruses with a single-stranded structure, classified under the genus Parechovirus in the family Picornaviridae [1]. The genus comprises six species (A–F), with PeV-A having 19 distinct genotypes. PeV-A3 is frequently identified in pediatric patients, as shown by phylogenetic analysis of the viral protein 1 (VP1) region [1, 2].

Infections from PeV-A3 usually present as mild or asymptomatic in healthy people, but they can result in serious complications in neonates and young infants. The clinical issues associated with PeV-A3 infection include sepsis, meningoencephalitis, hemophagocytic lymphohistiocytosis (HLH), and sepsis-like symptoms [3–6]. In vulnerable groups like neonates and young infants, differentiating severe PeV-A3 infection from other illnesses, including bacterial sepsis, poses clinical challenges.

The exact mechanisms behind PeV-A3 infection remain unclear. It is believed that direct cytopathic effects of the virus and the host's immune response play roles in the disease's severity [7, 8]. Neonates and infants whose mothers have lower antibody levels seem more likely to develop severe PeV-A3 infection [7]. Additionally, hypercytokinemia plays a role in the pathophysiology of severe cases, indicating that an overactive inflammatory response could worsen clinical outcomes [3–9]. Hyperferritinemia is a key laboratory observation in these infections, frequently seen during the acute phase. The involvement of ferritin in the immune response and its link to cytokine release is still under thorough investigation. However, the treatment of patients experiencing hyperferritinemia (hypercytokinemia) is not well established [10, 11].

We present a case involving a 25-day-old girl who experienced neonatal sepsis caused by PeV-A3, accompanied by considerable hyperferritinemia. Interestingly, the infant healed without requiring immunosuppressive treatment, emphasizing the possibility of favorable results even in severe PeV-A3 infection.

## 2. Case Presentation

A previously healthy female infant, 25 days old, was admitted to our hospital with a history of high fever, a growling voice, and poor feeding that began on the first day of her illness. She was born at full term via uncomplicated vaginal delivery. Maternal vaginal swab cultures during pregnancy were negative for *Streptococcus agalactiae*. The infant had been exclusively breastfed. Additionally, the patient's 4-year-old sibling had shown symptoms of cough and nasal discharge a few days earlier.

**Figure 1:**
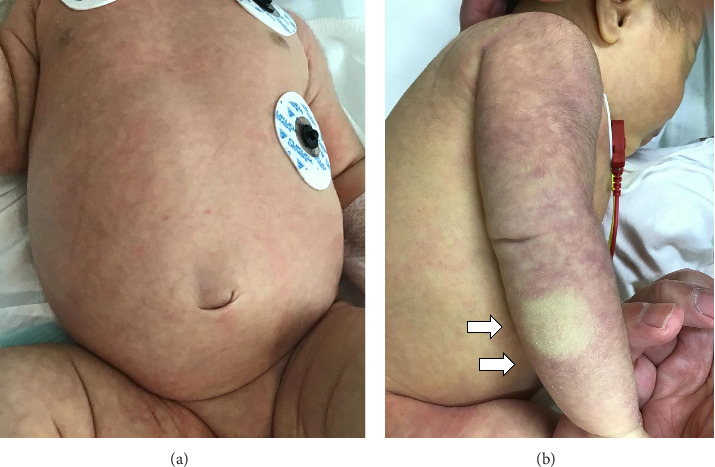
President neonatal sepsis caused by human parechovirus type A3 with marked hyperferritinemia characterized by (a) abdominal distension and (b) mottled skin across the infant's upper extremities. White arrows indicate marked prolongation of capillary refilling time.

The neonate was found to be lethargic during physical examination, scoring E2V3M4 on the Modified Glasgow Coma Scale. Her vital signs included a temperature of 39.2°C, blood pressure at 64/38 mmHg, heart rate of 187 beats per minute, respiratory rate of 60 breaths per minute, and oxygen saturation at 100% on room air. The infant displayed tachypnea, tachycardia, and grunting respirations, yet there were no indications of nasal flaring, intercostal or subcostal retractions, or reduced breath sounds. Abdominal distension was observed without umbilical protrusion (Figure 1(a)). Moreover, the neonate showed mottled skin and jaundice (Figure 1(b)). The capillary refill time was noted to be prolonged at 6 s.

Laboratory results indicated the following: a white blood cell count of 5600 cells/μL (normal range: 4500–13,500 cells/μL) with 62.5% neutrophils; a red blood cell count of 4.66 × 10^6^ cells/μL (normal range: 3.76 × 10^6^–5.00 × 10^6^ cells/μL); a hemoglobin level of 14.8 g/dL (normal range: 11.3–15.2 g/dL); hematocrit at 42.9% (normal range: 33.4%–44.9%); and a platelet count of 23.9 × 10^4^/μL (normal range: 13.1 × 10^4^–36.9 × 10^4^ cells/μL). Inflammatory biomarkers exhibited no elevation: C-reactive protein was 0.138 mg/dL (normal range: < 0.20 mg/dL), procalcitonin was 0.11 ng/mL (normal range: < 0.5 ng/mL), total bilirubin was 9.27 mg/dL (normal range: 0.20–1.20 mg/dL), direct bilirubin was 0.63 mg/dL (normal range: 0.05–0.30 mg/dL), lactate dehydrogenase (LDH) was 251 U/L (normal range: 120–240 U/L), aspartate aminotransferase (AST) was 30 U/L (normal range: 3–38 U/L), and alanine aminotransferase (ALT) was 11 U/L (normal range: 4–44 U/L).

Conversely, cytokine markers showed significant elevation, with ferritin measured at 2346 ng/mL (normal range: 4.6–204 ng/mL), soluble interleukin-2 receptor (sIL-2R) at 1932 U/mL (normal range: 590–1712 U/mL), and urine β2-microglobulin at 14,378 μg/L (normal range: < 289 μg/L). Serum cytokines were also notably increased: interleukin-1β (IL-1β) at 1.4 pg/mL (normal range: unavailable), tumor necrosis factor-α (TNF-α) at 2.4 pg/mL (normal range: < 1.1 pg/mL), interferon-γ (IFN-γ) at 7.8 pg/mL (normal range: < 0.5 pg/mL), interleukin-6 (IL-6) at 70.3 pg/mL (normal range: < 7.0 pg/mL), and interleukin-10 (IL-10) at 29 pg/mL (normal range: < 8 pg/mL). In cerebrospinal fluid (CSF), cytokine levels were generally not significantly elevated, except for IL-6, which was found at 17.6 pg/mL (normal range: < 7.0 pg/mL). Other CSF markers, including IL-1β, TNF-α, and IFN-γ, remained unchanged.

A septic workup was quickly started, encompassing blood, urine, CSF cultures, and viral studies. Analysis from a lumbar puncture showed normal levels of CSF protein and glucose, with no signs of pleocytosis. The CSF FilmArray Meningitis/Encephalitis polymerase chain reaction (PCR) panel (BioFire Diagnostics, Salt Lake City, Utah) tested positive for PeV. Real-time PCR examination of the blood and CSF samples confirmed the presence of PeV genomic RNA, yielding threshold cycle values of 21.76 for blood and 33.46 for CSF [12]. Further genotyping of PeV revealed the strain as PeV-A3 through sequencing of the VP1 region [2]. Testing for enterovirus was negative for both blood and CSF samples.

**Figure 2:**
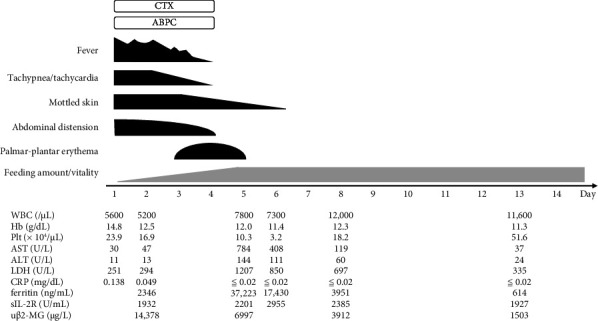
Clinical course of neonatal sepsis caused by human parechovirus type A3 with marked hyperferritinemia.

Following the clinical findings and laboratory results, a diagnosis of sepsis caused by PeV-A3 was confirmed. After collecting two sequential blood culture samples from the peripheral vein, empiric antimicrobial treatment with cefotaxime (200 mg/kg/day) and ampicillin (200 mg/kg/day) started on Day 1 of symptoms (Figure 2). The reddening of the palms and soles began on Day 3 following the fever and lasted 2 days.

On Day 4 of illness, her fever subsided. Her abdominal distension showed gradual resolution. We confirmed that blood, CSF, and urine cultures were negative, leading to the discontinuation of antibiotics. However, on Day 5, lab tests showed a drop in platelet count (10.3 × 10^4^/μL), a significant rise in liver enzymes (AST: 784 U/L, ALT: 144 U/L, LDH: 1207 U/L), and severe hyperferritinemia (ferritin: 37,223 ng/mL). Nevertheless, the patient's clinical condition, including her feeding ability and overall well-being, continued to improve. Since the patient experienced only a single cytopenia and showed no signs of clinical deterioration, we opted not to administer immunosuppressive therapy, including corticosteroids or intravenous immunoglobulin. The patient was closely monitored, and her lab values gradually returned to normal. She was discharged on Day 14 of her illness and fully recovered. At the time of discharge, there were no indications of altered consciousness, motor deficits, or sensory dysfunction. At one year of age, the patient achieved expected psychomotor developmental milestones and exhibited age-appropriate growth parameters. Ophthalmologic and audiologic assessments were within normal limits.

## 3. Discussion

We report a case of neonatal sepsis caused by PeV-A3, complicated by hyperferritinemia, without the development of HLH. Recent studies have increasingly recognized PeV-A3 as a significant cause of neonatal sepsis. Infections often present with nonspecific symptoms, including fever, lethargy, poor feeding, and respiratory distress. Understanding the clinical features and disease course is crucial for the early diagnosis of PeV infections.

Yuzurihara et al. detailed a group of nine neonates and infants affected by PeV-A3 infection, noting a median onset age of 31 days. In these instances, fever lasted for a median duration of 4 days (range: 3–5) [6]. In a similar study, 15 neonates and young infants were reported to have PeV-A3 infection, with a median age of 33 days (range: 10–81) and a median fever duration of 3 days (range: 1–4) [13]. In our case, the fever lasted for 4 days, aligning with previous reports. However, it is important to remember that fever alone is a nonspecific symptom and cannot directly indicate a diagnosis of PeV-A3 infection.

In clinical settings, when virologic testing might not be easily accessible, identifying key clinical signs can assist in diagnosing PeV-A3 infection. Various unique clinical features have been proposed as possible diagnostic markers of PeV-A3 infection, which include mottled skin, abdominal distention, palmar–plantar erythema, and hyperferritinemia [6, 13–16].

In our case, mottled skin appeared on Day 1 after the onset of fever and persisted for 4 days. Blotchy skin is considered an early sign of PeV infection but can also indicate peripheral circulatory failure. Therefore, differential diagnoses, including dehydration, bacterial infection, heart disease, and metabolic disorders, must be considered and ruled out.

Shoji et al. noted that 80% of their cohort (12 out of 15 patients) exhibited a unique palmar–plantar erythematous rash, which generally subsided [13]. In our instance, palmar–plantar erythema emerged on the third day following the onset of fever and lasted for 2 days. While Yeom et al. suggested that this palmar–plantar erythema could indicate clinical improvement in PeV-A3 infections, the underlying pathogenesis of this unique rash remains unknown [17].

PeV-A3 infections frequently start with vague symptoms such as fever and decreased appetite, lasting approximately 3 days. Alongside palmar–plantar erythema, important indicators like abdominal distension and navel protrusion might also occur, though these signs are not uniformly reported. Our observation noted abdominal distension without navel protrusion on Day 1.

Laboratory results for PeV-A3 infections frequently show increased ferritin, sIL-2R, urinary β2-microglobulin, and LDH levels, alongside decreased leukocyte and platelet counts, indicating the presence of hypercytokinemia [3, 14, 18]. Ferritin, an acute-phase reactant, is an inflammation and tissue damage marker. The increase in ferritin levels during PeV-A3 infection likely indicates a severe inflammatory response triggered by viral replication and immune activation. Hara et al. found that the ferritin levels in patients infected with PeV-A3 (mean: 2437 ng/mL) were considerably higher compared with those infected with other pathogens, such as enterovirus (mean: 552 ng/mL) or respiratory syncytial virus (mean: 237 ng/mL) [14]. Elevated ferritin levels typically appear 4-5 days following the onset of fever [14]. Hyperferritinemia and low platelet count were notably highest on Day 4 postfever onset, aligning with the patient's clinical improvement. This difference between clinical symptoms and lab results matches earlier findings, where laboratory parameters typically adjust later than clinical indicators. Additionally, prior research has indicated that hyperferritinemia associated with PeV-A3 infections is generally self-limiting and resolves without requiring immunosuppressive treatment therapy [18].

Shimizu et al. explored the kinetics of proinflammatory cytokines in PeV-A3-induced sepsis-like syndrome, revealing a strong association between IFN-γ and TNF-α with its development [19]. Our study noted heightened cytokine levels in the serum, while no significant rise was detected in the CSF. Furthermore, we observed increased IL-10 production, crucial in preventing HLH [20]. This could clarify why HLH did not progress, even with hyperferritinemia present.

We reviewed reported instances of PeV-A3 infection accompanied by hyperferritinemia (ferritin levels > 500 ng/mL) from January 2004 to December 2024, discovering 11 cases, including ours (Table 1). Sepsis or sepsis-like syndrome was the most frequent presentation, observed in 100% (11/11) of the cases, yet none fulfilled the diagnostic criteria for HLH. Regarding treatment, antimicrobial agents were administered to 4 cases: immunoglobulin to 1, steroids to 2, and cyclosporine to 1. Notably, 8 cases (73%) experienced spontaneous remission without requiring immunosuppressive therapy.

Elevated ferritin levels in PeV-A3 infections have highlighted the potential benefits of immunosuppressive therapies, such as corticosteroids, for managing the inflammatory response. Some studies indicate that steroids may alleviate cytokine storms and enhance clinical outcomes [6, 19, 21]. Limited evidence supports the regular use of immunosuppressive therapy. Patients showed improvement without these treatments, including the present case. Considering the risks of immunosuppressive therapy, such as the chance of exacerbating infections or secondary complications, its administration must be cautiously tailored to individual clinical situations.

Management of PeV-A3 infection remains supportive in the absence of effective antiviral treatments. Some suggest intravenous immunoglobulin (IVIG) may benefit severe cases based on its high neutralizing antibody titers [7]. However, Westerhuis et al. indicated that most PeV-A3 strains may not be effectively neutralized by IVIG, leaving its use uncertain [11]. In our case, we did not administer IVIG for these reasons.

Monitoring both clinical symptoms and laboratory values closely is crucial, especially when hypercytokinemia is present, to detect signs of organ failure or deterioration. Even if fever decreases and skin symptoms like mottled skin and palmar–plantar erythema improve, ferritin levels can continue rising. Recognizing the typical progression of PeV-A3 infection is essential for distinguishing it from potential complications. Should the clinical course veer from the anticipated path, further investigations must be conducted to uncover any issues.

Thus, we recommend that treatments like corticosteroids or other immunosuppressive therapies hinge on a comprehensive evaluation of the patient's clinical status and lab findings rather than just the extent of hyperferritinemia. Furthermore, more research is needed on the role of cytokine profiles in forecasting disease progression.

In summary, this instance of neonatal sepsis triggered by PeV-A3 resulted in marked hyperferritinemia without the onset of HLH. The infant showed improvement without administering corticosteroids or IVIG, reinforcing the notion that numerous PeV-A3 cases resolve independently. This indicates that not every PeV-A3 infection with hyperferritinemia necessitates immunosuppressive treatment. It underscores the necessity for thorough clinical and laboratory evaluations, emphasizing a cautious approach to immunosuppressive use.

## Figures and Tables

**Table 1:** Clinical and laboratory findings associated parechovirus type 3 infection with hyperferritinemia.

Case	Age (days)	Sex	Diagnosis	Day of defervescence	Ferrtin (day of disease onset)	Antimicrobial agent	Immunosuppressive therapy	Outcome	Literature
1	14	M	Sepsis/sepsis-like	3	2795 (N/A)	None	None	Cure	6
2	30	M	Sepsis/sepsis-like	3	699 (N/A)	None	None	Cure	6
3	31	M	Sepsis/sepsis-like	5	2511 (N/A)	None	DEX	Cure	6
4	43	F	Sepsis/sepsis-like	4	2104 (N/A)	None	None	Cure	6
5	38	M	Sepsis/sepsis-like	4	897 (N/A)	None	None	Cure	6
6	28	M	Sepsis/sepsis-like	5	22,383 (N/A)	ABPC, CTX, ACV	PSL, DEX, CyA	Cure	6
7	35	M	Sepsis/sepsis-like	6	31,679 (4)	None	None	Cure	16
8	38	F	Sepsis/sepsis-like	5	47,710 (4)	None	None	Cure	16
9	42	M	Sepsis/sepsis-like	N/A	2581 (5)	SBT/ABPC, CTX	IVIG	Cure	17
10	25	F	Sepsis/sepsis-like	6	630 (5)	SBT/ABPC, GM	None	Cure	17
11	25	F	Sepsis/sepsis-like	4	37,223 (4)	ABPC, CTX	None	Cure	Our case

*Note:* Each value denotes the worst one observed during the hospital course. ABPC, ampicillin; ACV, aciclovir; CTX, cefotaxime; CyA, cyclosporine A; DEX, dexamethasone; GM, gentamycin; IVIG, intravenous immunoglobulin; IVMP, intravenous methylprednisolone; SBT/ABPC, sulbactam/ampicillin; PSL, prednisolone.

Abbreviation: N/A, not available.

## Data Availability

Data sharing is not applicable to this article as no datasets were generated or analyzed during the current study.

## References

[1] Olijve L., Jennings L., Walls T. (2018). Human Parechovirus: An Increasingly Recognized Cause of Sepsis-Like Illness in Young Infants. *Clinical Microbiology Reviews*.

[2] Ito M., Yamashita T., Tsuzuki H. (2010). Detection of Human Parechoviruses from Clinical Stool Samples in Aichi, Japan. *Journal of Clinical Microbiology*.

[3] Aizawa Y., Izumita R., Saitoh A. (2017). Human Parechovirus Type 3 Infection: an Emerging Infection in Neonates and Young Infants. *Journal of Infection and Chemotherapy*.

[4] Marchand S., Launay E., Schuffenecker I., Gras-Le Guen C., Imbert-Marcille B. M., Coste-Burel M. (2021). Severity of Parechovirus Infections in Infants Under 3 Months of Age and Comparison With Enterovirus Infections: A French Retrospective Study. *Archives de Pediatrie*.

[5] Pietrasanta C., Ronchi A., Bassi L. (2024). Enterovirus and Parechovirus Meningoencephalitis in Infants: A Ten-Year Prospective Observational Study in a Neonatal Intensive Care Unit. *Journal of Clinical Virology*.

[6] Yuzurihara S. S., Tanaka F., Kai S. (2013). Human Parechovirus-3 Infection in Nine Neonates and Infants Presenting Symptoms of Hemophagocytic Lymphohistiocytosis. *Journal of Infection and Chemotherapy*.

[7] Aizawa Y., Watanabe K., Oishi T., Hirano H., Hasegawa I., Saitoh A. (2015). Role of Maternal Antibodies in Infants with Severe Diseases Related to Human Parechovirus Type 31. *Emerging Infectious Diseases*.

[8] Lin G. L., McGinley J. P., Drysdale S. B., Pollard A. J. (2018). Epidemiology and Immune Pathogenesis of Viral Sepsis. *Frontiers in Immunology*.

[9] Brisca G., Bellini T., Pasquinucci M. (2024). Clinical Course and Peculiarities of Parechovirus and Enterovirus Central Nervous System Infections in Newborns: A single-center Experience. *European Journal of Pediatrics*.

[10] Vandenhaute J., Wouters C. H., Matthys P. (2020). Natural Killer Cells in Systemic Autoinflammatory Diseases: A Focus on Systemic Juvenile Idiopathic Arthritis and Macrophage Activation Syndrome. *Frontiers in Immunology*.

[11] Westerhuis B. M., Koen G., Wildenbeest J. G. (2012). Specific Cell Tropism and Neutralization of Human Parechovirus Types 1 and 3: Implications for Pathogenesis and Therapy Development. *Journal of General Virology*.

[12] Nix W. A., Maher K., Johansson E. S. (2008). Detection of all Known Parechoviruses by real-time PCR. *Journal of Clinical Microbiology*.

[13] Shoji K., Komuro H., Miyata I., Miyairi I., Saitoh A. (2013). Dermatologic Manifestations of Human Parechovirus Type 3 Infection in Neonates and Infants. *The Pediatric Infectious Disease Journal*.

[14] Hara S., Kawada J., Kawano Y. (2014). Hyperferritinemia in Neonatal and Infantile Human parechovirus-3 Infection in Comparison with Other Infectious Diseases. *Journal of Infection and Chemotherapy*.

[15] Shoji K., Komuro H., Kobayashi Y. (2014). An Infant with Human Parechovirus Type 3 Infection With a Distinctive Rash on the Extremities. *Pediatric Dermatology*.

[16] Casas-Alba D., Martínez-Monseny A., Monfort L. (2016). Extreme Hyperferritinemia in Dizygotic Twins With Human Parechovirus-3 Infection. *The Pediatric Infectious Disease Journal*.

[17] Yeom J. S., Park J. S., Seo J. H. (2016). Distinctive Clinical Features of HPeV-3 Infection in 2 Neonates With a Sepsis-like Illness. *Korean J Pediatr*.

[18] Nimura K., Maruyama Y., Aizawa Y., Saitoh A., Nakazawa Y. (2020). Changes in Laboratory Findings in Parechovirus-A Infection in Nine Neonates and Infants. *Pediatrics International*.

[19] Shimizu M., Shimizu H., Jinkawa A. (2020). Cytokine Profiles in Human Parechovirus Type 3-Induced Sepsis-Like Syndrome. *The Pediatric Infectious Disease Journal*.

[20] Behrens E. M., Canna S. W., Slade K. (2011). Repeated TLR9 Stimulation Results in Macrophage Activation Syndrome-Like Disease in Mice. *Journal of Clinical Investigation*.

[21] Sen E. S., Ramanan A. V. (2024). Cytokine Storm Syndrome Associated with Hemorrhagic Fever and Other Viruses. *Advances in Experimental Medicine and Biology*.

